# Broadening the Use of the Concealed Information Test in the Field

**DOI:** 10.3389/fpsyt.2019.00024

**Published:** 2019-02-05

**Authors:** Izumi Matsuda, Tokihiro Ogawa, Michiko Tsuneoka

**Affiliations:** National Research Institute of Police Science, Tokyo, Japan

**Keywords:** concealed information test (CIT), statistical discrimination, field application, memory detection, searching CIT

## Abstract

Japan is the only country where the polygraph with the concealed information test (CIT) is widely applied to criminal investigations. The CIT can reveal whether an examinee has knowledge of specific details of a crime. Furthermore, the CIT can extract crime-relevant information that investigative organizations have not yet uncovered. This article introduces how Japanese polygraphers take advantage of the CIT in criminal investigations. We also describe how polygraphs with the CIT are currently used in court. Then we propose statistical discrimination methods that can be easily applied to CIT interpretation in the field. Appropriate application of the statistical values is discussed. We hope that this article will facilitate more active use of the CIT outside Japan.

Many people regard the polygraph as a deception detection technique. However, the polygraph using the concealed information test (CIT) does not aim to detect deception: rather, it aims to detect crime-relevant memory. The CIT can assess whether an examinee knows details of a crime, despite saying “I don't know.” The CIT also can provide clues about crime details that the investigative organization has not yet grasped. However, despite its effectiveness, the CIT is widely used only in Japan. In this article, we aim to address this situation and facilitate more active use of the CIT. We first introduce how Japanese polygraphers take advantage of the CIT. We then propose simple scoring methods and their possible thresholds, which can be easily applied in the field.

## Polygraph as a Memory Detection Test

The term *polygraph* generally refers to a test conducted with a polygraph device. In forensic situations, a polygraph measures autonomic responses to questions related to a crime. Autonomic responses, such as skin conductance and respiration, have high signal-to-noise ratios and can easily be measured outside controlled laboratory settings, unlike central measures such as electroencephalograms (1). Thus, in the field of criminal investigations, autonomic responses are still preferred to central responses (2).

There are several question techniques for the polygraph. Worldwide, the most commonly used technique is the control question test or comparison question test (CQT) (3). In the CQT, an examiner asks crime-relevant questions (e.g., “Did you rob the Mart last night?”), comparison questions (e.g., “Did you ever take something that did not belong to you?”), and neutral questions (“Did you live in the United States?”). The CQT aims to reveal whether an examinee has lied about the crime-relevant question by comparing the physiological responses for the crime-relevant and comparison questions.

The CIT, or the guilty knowledge test, is another question technique for the polygraph, although it does not directly aim to detect deception. The CIT assesses the examinee's memory of a particular crime detail (4, 5). For a question about the crime detail (e.g., the accessory that was stolen from the Mart), the examiner typically shows five items as possible answers (e.g., “a necklace?” “an earring?” “a watch?” “a brooch?” “a ring?”), including one correct (i.e., actually crime-relevant) item. These items are selected so that persons who do not know the crime detail cannot distinguish the crime-relevant item from the irrelevant items. The perpetrator can distinguish the crime-relevant item, but may attempt to avoid revealing this to the examiner, to conceal his or her involvement in the crime. Therefore, the CIT is conducted when the examinee claims that he or she does not know which is the crime-relevant item among the items. The examiner infers that the examinee in fact recognizes the crime-relevant item, despite his or her statement to the contrary, when the responses to the crime-relevant item differ from those to the crime-irrelevant items. Typically, greater skin conductance, suppressed respiration, slower heart rate, and smaller pulse volume are observed for the relevant item than for the irrelevant items [for reviews, see (1, 6)].

The validity of the CIT has been confirmed by laboratory studies. Elaad (7) conducted a meta-analysis of laboratory CIT studies and found that the weighted average of the false positive rates was 4.1%, and that of the false negative rates was 19.4%. A recent meta-analysis showed discrimination performance of each measure: the areas under the receiver operating characteristic (ROC) curve of skin conductance response, respiration, and heart rate were 0.848, 0.770, and 0.735, respectively (8). The CIT has been found to achieve high discrimination performance, with particularly low false positive rates (9).

## The CIT in Japan

Despite the validity of the CIT described above, it is rarely used in real criminal investigations worldwide. One potential reason is that many practitioners have not known how to apply the CIT in the field. In this section, we introduce the field use of the CIT in Japan, where the CIT has been widely used for criminal investigations.

In Japan, the CIT is the only polygraph application used in criminal investigations. The CQT is not currently used at all. About 100 polygraph examiners deal with about 5,000 cases per year (10). These examiners administer the polygraph after completing a 3 month training course at the Forensic Science training center, affiliated with the National Research Institute of Police Science.

**Figure 1:**
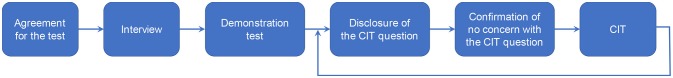
Flowchart of the polygraph in Japan.

Figure 1 outlines how the polygraph is conducted in Japan. A consenting examinee receives the polygraph. At the beginning of the test, the examiner interviews the examinee to check what the examinee says about his or her knowledge of the crime. If the examinee says that he or she knows some crime details, the examiner will not perform CITs on these details.

Then the examiner attaches sensors to the examinee. In Japan, the examiner usually records several physiological measures: an electrocardiography (ECG), respiratory movement, skin conductance, and pulse wave. The ECG is used for computing heart rate. The pulse waves recorded with different filter settings are used for computing the normalized pulse volume (11).

The examiner conducts a so-called card test as a demonstration of a CIT. Typically, the examinee is asked to select one playing card from several playing cards with different numbers (e.g., 3, 4, 5, 6, and 7) and to memorize the number on it. Then the examiner asks the examinee which number he or she selected by presenting the numbers one by one, with an inter-stimulus interval of about 20–30 s. This process shows the examinee how the following CITs will be conducted. Additionally, through this card test the examiner can observe how the examinee physiologically responds to the item that he or she recognized.

Next, the examiner conducts the CITs. One CIT question usually consists of four to six items, one of which is supposed to be related to the crime. Before conducting the CIT, the examiner shows the examinee the CIT question and all included items and confirms the following three points. First, whether the examinee understands the meaning of the question and the items. If the examinee seems to have trouble with understanding the question or the items, the examiner adds explanations or replaces words with easier ones. Second, whether the examinee claims to know which item is crime-relevant. If the examinee says, prior to the test, that he or she can identify the crime-relevant item, the examiner does not conduct the test for that question. Finally, whether the examinee says that he or she is concerned about any items. For example, in the above CIT on the stolen accessory, if an examinee bought a watch a few days before, he may show a large response to the item “watch,” even though the examinee has no crime-related knowledge. If the examinee says that he or she is concerned about a certain item, the examiner often replaces it to another item or discards the question.

In the CIT, the examiner vocally, and sometimes visually, presents each item, with the inter-stimulus interval of about 20–30 s. After all items have been presented, a short break is inserted if needed. This process is usually repeated 3–5 times, changing the order of the items to remove possible confounding effects due to the presentation order. After the CIT, the examiner often asks to the examinee whether he or she has any concerns about the test.

Based on the responses to the items, the examiner examines whether responses to a specific item are different from those to other items. If the examiner observes differences in responses between items, the examiner will infer that the examinee recognizes a specific item as crime-relevant.

Typically, the examiner conducts 4–7 CIT questions (12), each of which deals with different crime-relevant information. For example, in a theft case, in addition to the CIT on the stolen item, the examiner may conduct CITs on the time the crime happened, the crime scene, and the placement of the stolen item at the scene.

## What the CIT Can Reveal in Criminal Investigations

As described above, the CIT examines whether the examinee recognizes a crime-relevant item that only a person associated with the crime could possibly know. More concretely, the CIT is conducted in Japan (1) to reveal whether the examinee knows a specific criminal detail, (2) to obtain new crime-relevant information, and (3) to reveal whether the examinee's statement is true.

### Whether the Examinee Knows a Criminal Detail

This is the most typical usage of the CIT, an example of which is described in section Polygraph as a Memory Detection Test. Consider that there a crime-relevant fact has been obtained through an investigation (e.g., a ring was stolen). If it is assumed that only a person related to the crime could know this crime-relevant fact, the CIT can be used to examine whether the examinee does indeed know the fact. If the CIT result indicates that the examinee knows the fact, the investigators will extend the investigation to reveal the reason (e.g., because the examinee committed the theft or was an accomplice).

### New Crime-Relevant Information

The CIT also can reveal crime-relevant information that even investigative organizations have not yet discovered. This type of the CIT is called a searching CIT. The searching CIT is conducted in the same way as the usual CIT. However, in the searching CIT, the examiner does not know which item is crime-relevant. For example, consider a case that a woman is missing. In this case, the examiner might conduct a CIT on the woman's location. The examiner may ask “Is she in City A? City B? City C? City D? City E? Another city?” to the examinee and compare responses among items. If the responses differ between City C and other items, the examiner infers that the examinee knows that she is in City C. In this case, the investigators can focus their search on City C to find her. In this way, the result of the searching CIT can be used to find new evidence and streamline investigations. Osugi (10, 13) reported other practical examples in which the searching CIT has been applied.

### Credibility of the Examinee's Statement

The CIT also can be used to infer whether the examinee's statement is true or not. Osugi (13) reported this example: an examinee who sold a stolen ring insists that he found the ring on the road. To determine whether this statement is true, the examiner can conduct a CIT consisting of other possibilities (e.g., “You received the stolen ring from someone without paying anything,” “You paid money to get the stolen ring,” “You stole the ring yourself and did it alone,” “You stole the ring together with an accomplice,” “You got the ring in some other way”). If differential responding is not observed for any items, the examinee's statement that he found the ring on the road would be evaluated as true. In contrast, if differential responding is observed for a specific item in the CIT, his statement would be considered false. The CIT can assess not only whether the statement is true, but also what the truth is, as the examinee remembers it. This type of CIT also can be used to examine eyewitness or victim statements. However, few research has been conducted on this topic; future research is expected to support this usage of CIT.

### The Difference Between Laboratory and Field CIT

As shown above, the CIT is used in the field in Japan to reveal examinees' recognition of the details of a crime. This approach differs from that used in typical laboratory CIT studies, which usually integrate responses among all CIT questions and conclude whether the examinee is guilty or innocent (14–16). Ben-Shakhar and Elaad (17) reported that discrimination performance was much higher for integrating responses from 12 different CIT questions repeated once, than for integrating responses from one CIT question repeated 12 times.

However, in the field, it is sometimes difficult to find enough crime details that have not been publicly announced. Thus, Japanese examiners actively use the searching CIT (10). Since the crime-relevant item is not identified in the searching CIT, integrating multiple CIT questions is impossible.

Moreover, it is difficult to assume that a person relevant to a crime remembers all the details. He or she may forget or genuinely not know some details. For example, the CIT in a theft case may reveal that the examinee knows the time the crime happened and the crime scene, but does not recognize the placement of the stolen item at the scene. This suggests the possibility that the examinee only drove a perpetrator to the crime scene.

Analyzing CIT questions individually can reveal what the examinee knows and what he or she does not know about the crime. Such an approach is sometimes much more informative in criminal investigations than integrating the CIT questions to conclude whether the examinee is guilty or innocent. However, it should be noted that this approach requires a sufficient number of repetitions of each CIT question to maintain high discrimination performance (18, 19).

## CIT in Courts

In Japan, the results of the polygraph are usually used by investigative organizations as tools to assess whether and how the examinee is related to the crime. The results are rarely dealt with in court: a few of the about 5,000 cases are discussed each year. However, the Supreme Court admitted polygraph results as an evidence in 1968. Recent legal literature has noted that the probative value of the CIT result can be relatively high if the CIT is correctly conducted to examine the defendant's knowledge of facts that only the perpetrator could know (20). That is, the CIT result that the defendant knows the crime-relevant fact can be one reference information for the judge to decide whether he/she is guilty.

We checked court precedents relevant to the polygraph for the last 10 years. In many cases, legal professionals have focused on whether differential responding to the crime-relevant fact could be explained other than via a memory obtained through perpetration. For example:

- The defendant might have had an opportunity to encounter the fact through interrogation and rumors.
- The defendant might have had prior concerns about the fact because of personal reasons irrelevant to the crime.
- The defendant might have speculated about the fact.

These possibilities can detract from the probative value of the CIT for demonstrating the defendant's knowledge about the crime-relevant fact. As we mentioned above, the examiner conducts the CIT after confirming that the examinee says that he or she has no concerns about any of the items. The examiner should properly denote this confirmation process in the report.

In criminal investigations, the CIT can also be used to extract new information that the investigators had not previously known about (section New Crime-Relevant Information), and to examine the credibility of the examinee's statement (section Credibility of the Examinee's Statement). When differential responding is observed for a specific item in these CITs, later criminal investigations try to obtain new facts or statements underpinning the results. However, if such new facts or statements are not obtained, these CIT results would be rarely discussed in court.

## A Remaining Task for the CIT in Japan

In Japan, the CIT has been widely used in criminal investigations and sometimes discussed in court. However, there are issues that remain to be solved. One issue is related to the process for assessing physiological differences. Below, we introduce the current judgment method in Japan and discuss statistical judgment in the following sections.

### Current Judgment Method in Japan

Japanese polygraphers primarily judge differences in autonomic responses by visual inspection. Osugi (10) explained this judgment process as follows: the examiner ascertains whether the examinee showed differential responses based on the charts, the difference between the mean responses to crime-relevant and irrelevant items, and the consistency of the response differences across repetitions. It has been repeatedly confirmed that the discrimination performance of this judgment is sufficiently high (21–23). The latest study was conducted by Ogawa et al. (23), where 36 Japanese polygraphers blindly judged experimental CIT data from 152 examinees by visual inspection. Eighty examinees performed a mock crime before the CIT, while 72 examinees did nothing. Of the cases, 20.4% were judged as inconclusive. Excluding the inconclusive cases, the hit rate was 86.4%, and the correct rejection rate was 94.5%.

This high performance of visual inspection judgments could be attributable to its flexibility for inter- and intra-individual response differences. Which autonomic measures clearly respond to the relevant item differs across individuals (24). Furthermore, an examinee's reactivity can change between the first half and the second half of the polygraph, because of habituation and fatigue. Visual inspection enables the examiner to flexibly adjust the measures to consider the examinee's response tendency at that time.

However, visual inspection is sometimes regarded as subjective and dependent on the skill and experience of the examiner (3, 25). Introducing statistical judgment methods will make the CIT more objective and scientifically valid, even if the performance does not increase (2, 13). Increased objectivity will enhance the probative power of CIT results in court.

### Requirements of Statistical Methods for Field Use

Recently, researchers have proposed many statistical classification methods [(24, 26, 27) for a review, see (2)]. However, the chosen statistical method for interpreting CITs in the field should meet the following requirements.

1. Simplicity. The examiner may have to explain the judgment process in court. A simple method is required so that law and citizen judges can understand easily.
2. Low false positive rate. In criminal investigations, at least in Japan, attempts are made to avoid false charges as much as possible. Although the low likelihood of false positives constitutes a major advantage for the CIT (9), measures should be taken to minimize the occurrence of false positive cases, while maintaining the relatively small number of inconclusive and false negative cases.
3. Manageability for missing measures. In the field, the examiner sometimes cannot use some measures for analysis. For example, the rate of electrodermal non-responsivity is about 25% (28). A statistical method that can flexibly deal with such a situation is preferable.
4. Avoidance of database use. Autonomic responses are influenced by age, sex, season, time of day, and so on (28). A database that would be appropriate for all examinees is thus difficult to envision at present.

## Discrimination Based on Effect Size and Randomization

Considering the above four conditions, Matsuda et al. (29) proposed the use of the *d* value for effect size (30–32) and the *p*-value of the randomization test (33). Both *d* and *p*-values can be simply computed and require no database. In this section, we first explain how to compute *d* values (section Known-Solution CIT) and *p*-values (section Searching CIT). We then introduce the performances of *d* and *p*-values as compared with that of a traditional method (i.e., Lykken scoring) according to Matsuda et al. (29) (section Summary of the Threshold). We also compared these performance data with those of recent machine learning methods.

### Effect Size

Consider a CIT consisting of five items, each of which is presented five times, which measures heart rate, skin conductance, respiration, and normalized pulse volume. That is, the number of responses to the crime-relevant item is five (i.e., *n*_1_ = 5) and the number of responses to crime-irrelevant items is 20 (i.e., *n*_2_ = 20) for each measure. The difference between the mean of the responses to the crime-relevant item and the mean of the responses to the irrelevant items is divided by a standard deviation, which is the effect size *d*. The standard deviation for computing the effect size has several calculation methods (34). Here, we calculate the effect size *d* by the following pooled standard deviation (*s*_*p*_) using the unbiased variance of the responses to the relevant item (*s*${}_{1}^{2}$) and that to the irrelevant item (*s*${}_{2}^{2}$):

$$\begin{array}{l} s_{p}=\sqrt{\frac{\left(n_{1}-1\right)s_{1}^{\;2}+\left(n_{2}-1\right)s_{2}^{\;2}}{n_{1}+n_{2}-2}} \end{array}$$

In general, when the examinee recognizes the relevant item, the relevant item elicits greater skin conductance, but slower heart rate, depressed respiration, and smaller normalized pulse volume than the irrelevant items. Thus, *d*s of heart rate, respiration, and normalized pulse volume are multiplied by −1.

The effect size *d* is computed for each measure. To integrate the results of all measures, we simply average their *d* values so far. If some measures are missing, we can average the *d* values across the remaining measures.

### Randomization Test

**Figure 2:**
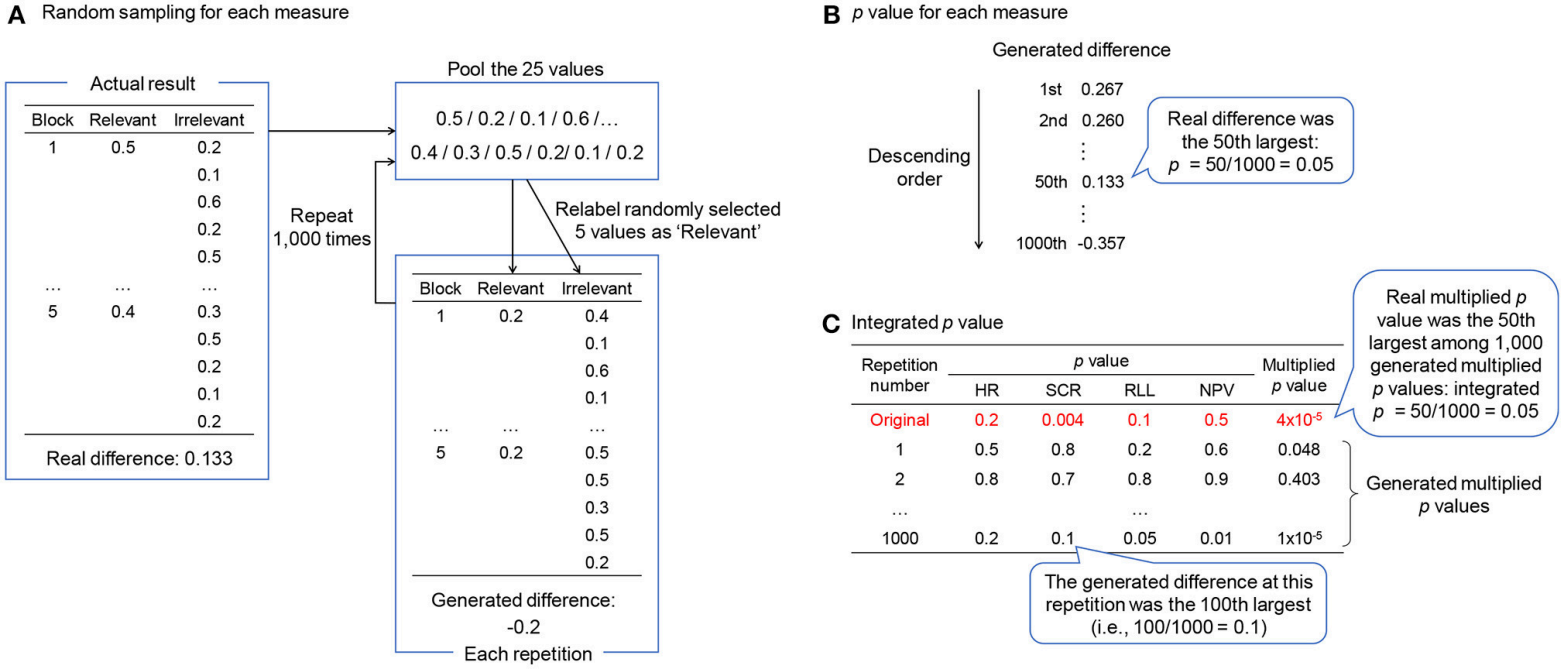
The procedure of the randomization test. **(A)** How to compute the generated response difference in each repetition. **(B)** How to compute the *p*-value for each measure. **(C)** How to integrate *p*-values across all measures. HR, heart rate; SCR, skin conductance response; RLL, respiration line length; NPV, normalized pulse volume.

The randomization test calculates the probability that the response difference between relevant and irrelevant items is obtained randomly. If the response difference can be obtained randomly, it means that we might obtain a similar response difference by randomizing the correspondence between the responses and the items. The procedure of the randomization test is shown in Figure 2. We assume a CIT consisting of five items × five repetitions and measuring heart rate, skin conductance, respiration, and normalized pulse volume. As shown in Figure 2A, five out of the 25 values for each measure are randomly selected and relabeled as the responses to the relevant item; the remaining 20 values are relabeled as the responses to the irrelevant items. Then the difference is computed between the mean of the values relabeled as relevant and the mean of the values relabeled as irrelevant. This process is repeated up to thousands of times (here, 1,000 times). Thus, we obtain 1,000 generated response differences. Regarding skin conductance, as shown in Figure 2B, if the real difference is the *x*th largest among the generated response differences, the *p*-value is calculated as *x*/1,000 (e.g., if *x* = 50, *p* = 0.05). Regarding heart rate, respiration, and normalized pulse volume, if the real difference is the *x*th smallest among the generated response differences, the *p*-value is calculated as *x*/1,000. Unlike the *t* test, the randomization test does not assume population parameters (35), which would be preferable for the CIT, whose sample size is rather small.

The method of integrating the results of each measure is shown in Figure 2C. At first, the *p*-value of each measure is multiplied across all measures. This is the original multiplied *p*-value. In contrast, we can calculate the *p*-value for each of the 1,000 repetitions by ranking the generated response difference at a certain repetition among 1,000 generated response differences. We then multiply these *p-*values across all measures. Thus, 1,000 multiplied *p-*values are generated. If the original multiplied *p-*value is the *x*th smallest among the generated multiplied *p*-values, the integrated *p*-value is *x*/1,000. If some measures are missing, we can multiply the *p*-values across the remaining measures.

### Performance of *d* and *p*

Matsuda et al. (29) assessed the performance of *d* and *p*-values using the dataset of Ogawa et al. (23). The dataset consists of experimental CIT data from 152 examinees. Eighty of the examinees stole a ring in a mock crime, and 72 did not. The CIT consisted of five accessory names, including “ring,” each of which was presented five times to examinees. During the CIT, respiration line length, skin conductance, heart rate, and normalized pulse volume were measured. For more details about the dataset, see Matsuda et al. (32), which is written in English.

Matsuda et al. (29) computed the integrated *d* and *p*-values for each CIT, in addition to the integrated Lykken score. Lykken score is a traditional scoring method (4) that assigns 2 to the largest response and 1 to the second-largest response in a block of repetitions, and then summarizes the scores across all blocks. The Lykken scores were integrated across all measures by averaging. The area under the ROC curve was 0.92, 0.92, and 0.90, for the integrated *d* value, the integrated *p*-value, and the integrated Lykken score, respectively. However, the ROC curve showed that maintaining a low false positive rate is more difficult for the integrated Lykken score than for the integrated *d* and *p*-values. This is probably because the Lykken score necessarily assigns scores even if the examiner observes no salient response in the block.

Recently, many machine learning methods have been proposed. We applied typical machine learning methods to the same dataset used in Matsuda et al. (29) using the Classification Learner App in MATLAB R2018a. This app automatically calculates the performance of various classifiers by protecting against overfitting using cross-validation. We computed the area under the ROC curve of decision trees, discriminant analysis, logistic regression, support vector machine, nearest neighbors, and ensemble classification. The area under the ROC curve was 0.85 (decision tree), 0.92 (discriminant analysis), 0.92 (logistic regression), 0.91 (support vector machine), 0.91 (nearest neighbors), and 0.92 (ensemble classification). The performances of the machine learning methods are almost the same as those of *d* and *p*-values. The calculation of *d* and *p*-values is simpler than these machine learning methods. Moreover, the machine learning methods require a database to estimate parameters, whereas the *d* and *p*-values do not. Thus, *d* and *p*-values are currently more useful for field CIT.

## Discrimination Threshold

As shown above, the performances of the effect size *d* and the randomization test *p* were sufficiently high. However, in the field, we should decide on thresholds for these statistical values to enable practitioners to judge whether the responses differ or not for each CIT. In this section, we show reference information for deciding thresholds in the case where the crime-relevant item is designated in advance and the case where it is unknown. We use the same dataset used by Ogawa et al. (23) described in section Performance of *d* and *p*: 80 recognizing and 72 unrecognizing examinees received the CIT with five items, which was presented five times.

### Known-Solution CIT

The known-solution CIT assesses whether an examinee recognizes the crime-relevant information that the investigative organization has already grasped. In this section, response differences between relevant and irrelevant items are scored as *d* or *p-*values.

#### *d* Value

**Figure 3:**
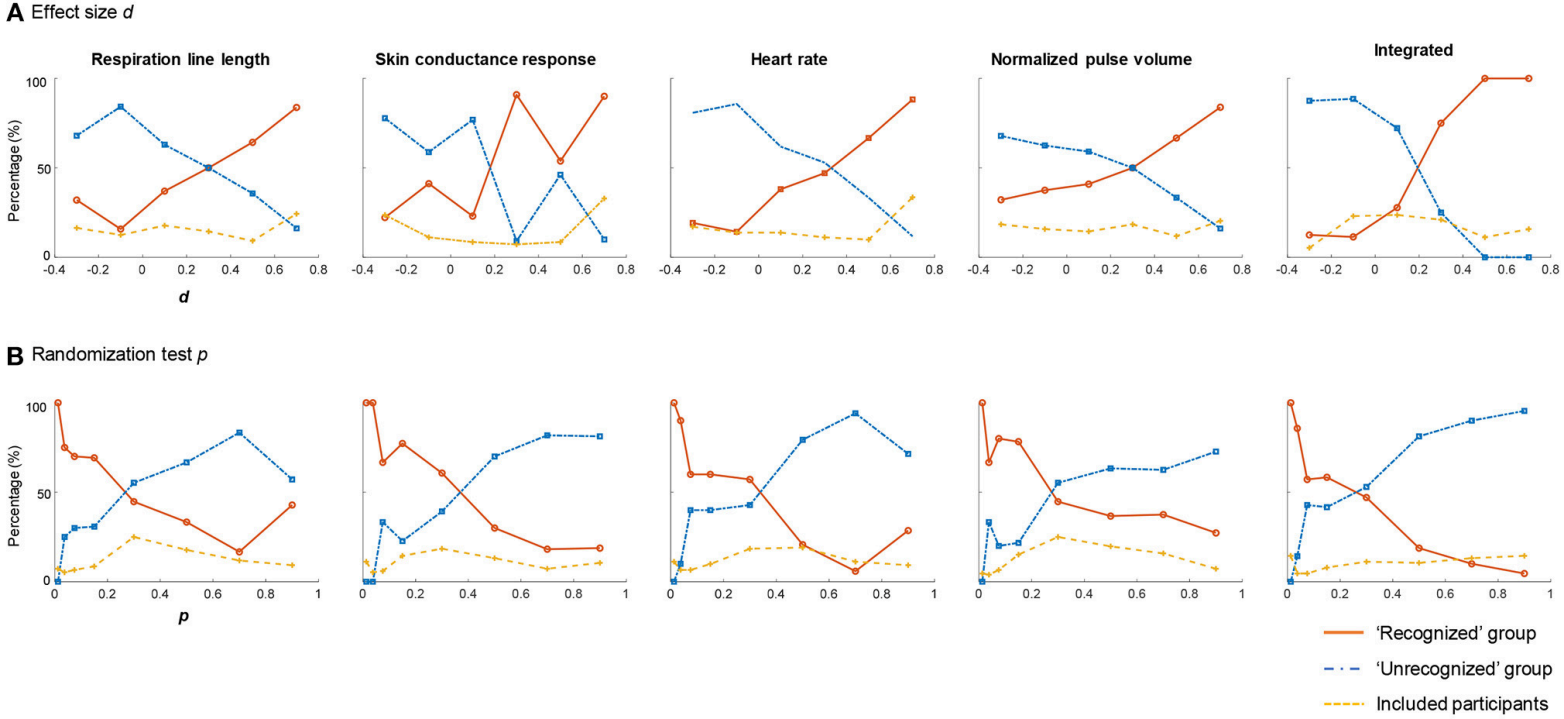
The statistical values for the known-solution CIT. **(A)** The percentage of the recognized or unrecognized examinees whose *d* values are in the range of < −0.2, −0.2–0, 0–0.2, 0.2–0.4, 0.4–0.6, or > 0.6, respectively. **(B)** The percentage of the recognized or unrecognized examinees whose *p*-values are in the range of 0–0.025, 0.025–0.05, 0.05–0.1, 0.1–0.2, 0.2–0.4, 0.4–0.6, 0.6–0.8, or 0.8–1, respectively. The solid red solid line shows (the number of recognizing examinees whose *d* or *p* scores are in each range)/(the number of examinees whose *d* or *p* scores are in each range). The blue chain line shows (the number of unrecognizing examinees whose *d* or *p* scores are in each range)/(the number of examinees whose *d* or *p* scores are in each range). The dashed yellow dash line shows (the number of examinees whose *d* or *p* scores are in each range)/(the number of all examinees).

Figure 3A shows the percentage of the recognizing and unrecognizing examinees whose *d* values of the CIT are in the range of < −0.2, −0.2–0, 0–0.2, 0.2–0.4, 0.4–0.6, or > 0.6, respectively^1^. The dashed yellow line shows the ratio of the examinees whose *d* scores are in each range to all examinees. The solid red line shows the ratio of the recognizing examinees to all examinees whose *d* scores are in each range. The blue chain line shows the ratio of the unrecognizing examinees to all examinees whose *d* scores are in each range.

Figure 3A shows that, for each measure, over 80% of examinees whose *d* values were > 0.6 did indeed have recognition of the relevant item. For heart rate, over 80% of the examinees whose *d* values were < 0 did not have recognition of the relevant item. As shown in the extreme right of Figure 3A, 100% of the examinees whose integrated *d* values were > 0.4 did indeed have recognition of the relevant item. More than 80% of the examinees whose integrated *d* values were < 0 did not have recognition of the relevant item.

If we judge the case of an integrated *d* > 0.4 as *recognized*, 0 < *d* < 0.4 as *inconclusive*, and *d* < 0 as *unrecognized*, the inconclusive rate is 44.7%. Without the inconclusive cases, the hit rate is 89.1% and correct rejection rate is 100%. We can reduce the inconclusive cases by judging the case of integrated *d* > 0.3 as *recognized*, 0.1 < *d* < 0.3 as *inconclusive*, and *d* < 0.1 as *unrecognized*. In this case, the inconclusive rate is 20.4%, the hit rate is 86.4%, and the correct rejection rate is 94.6%.

#### *p* Value

Figure 3B shows the percentage of the recognizing or unrecognizing examinees whose *p*-values are in the range of 0–0.025, 0.025–0.05, 0.05–0.1, 0.1–0.2, 0.2–0.4, 0.4–0.6, 0.6–0.8, or 0.8–1, respectively. The dashed yellow line shows the ratio of the examinees whose *p-*values are in each range to all examinees. The solid red line shows the ratio of the recognized examinees to all examinees whose *p*-values are in each range. The blue chain line shows the ratio of the unrecognized examinees to all examinees whose *p*-values are in each range.

As shown in Figure 3B, for each measure, 100% of the examinees whose *p-*values were < 0.025 did indeed have recognition of the relevant item. As shown in the extreme right of Figure 3B, more than 90% of the examinees whose integrated *p-*values were > 0.6 did not have recognition of the relevant items.

If we judge the case of the integrated *p* < 0.025 as *recognized*, 0.025 < *p* < 0.6 as *inconclusive*, and *p* > 0.6 as *unrecognized*, the inconclusive rate is 40.8%. Without the inconclusive cases, the hit rate is 94.1% and the correct rejection rate is 100%. If we want to reduce the inconclusive cases by judging the case of the integrated *p* < 0.05 as *recognized*, 0.05 < *p* < 0.4 as *inconclusive*, and *p* > 0.4 as *unrecognized*, the inconclusive rate is 24.3%, the hit rate is 88.5%, and the correct rejection rate is 98.2%.

Figure 3 also indicates that the integration of multiple measures dramatically improves the discrimination performance. The integrated *d* and *p*-values clarify the difference between the recognized and unrecognized groups and reduce the range judged inconclusive, where the percentages of the recognized and unrecognized examinees are competing.

### Searching CIT

In the searching CIT, an examiner assesses whether an examinee recognizes any of the items in a CIT question as crime-relevant. Thus, the examiner has to compare responses among all items. If the maximum response is sufficiently great and reliable, the examiner judges that the examinee recognizes the item as crime-relevant.

In this section, we examine the thresholds of the *d* and *p*-values for the searching CIT. We used the same dataset described in the above section but assume that the relevant item is unknown: we calculate five *d* or *p*-values for a CIT question assuming that each of the five items is the relevant item. We compare the five values to judge whether the examinee recognizes any item, and, if so, which item is recognized.

#### *d* Value

**Figure 4:**
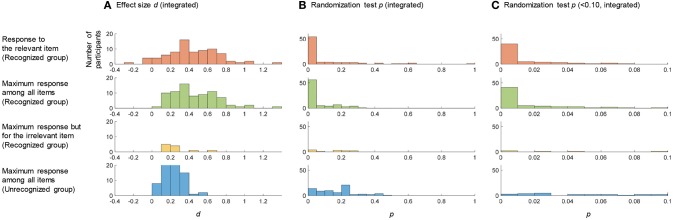
The statistical values for the searching CIT. The first panel shows the histogram of the integrated *d* **(A)** or *p* **(B,C)** for the actual relevant item in the recognized group. The second panel shows the histogram of the maximum/minimum integrated *d/p* among all items in the recognized group. The third panel shows the histogram of the integrated *d/p* that is maximum/minimum among all items but the item is actually irrelevant in the recognized group. The fourth panel shows the histogram of the maximum/minimum integrated *d/p* among all items in the unrecognized group.

Figure 4A shows histograms of the integrated *d* values for the searching CIT. The first panel shows the integrated *d* for the actual relevant item in the recognized group. The second panel shows the maximum integrated *d* among the five items in the recognized group. The third panel shows the maximum integrated *d* among the five items but where the item is actually irrelevant in the recognized group. The fourth panel shows the maximum integrated *d* among the five items in the unrecognized group. In the searching CIT, we must avoid two types of false positive cases: the case that recognizing examinees are judged as recognizing an irrelevant item, and the case that unrecognizing examinees are judged as recognizing a certain item. The threshold to avoid the first type of false positive is suggested by comparing the first panel with the third panel, and the threshold to avoid the second type of false positive is suggested by comparing the first panel with the fourth panel. Figure 4A shows that both types of false positive cases can be avoided by the threshold of 0.6. If we judge the case of the maximum integrated *d* > 0.6 as *recognized*, 0.2 < *d* < 0.6 as *inconclusive*, and *d* < 0.2 as *unrecognized*, the inconclusive rate is 54.6%, the hit rate is 63.9%, and the correct rejection rate is 100%.

#### *p* Value

Figures 4B,C show histograms of *p*-values for the searching CIT. The first panel shows the integrated *p* for the relevant item in the recognized group. The second panel shows the minimum integrated *p* among the five items in the recognized group. The third panel shows the minimum integrated *p* among the five items but where the item is actually irrelevant in the recognized group. The fourth panel shows the minimum integrated *p* among the five items in the unrecognized group. The comparison between the first panel and the third/fourth panel of Figure 4C reveals that we can avoid false positive cases with a threshold of 0.01. If we judge the case of the minimum integrated *p* < 0.01 as *recognized*, 0.01 < *p* < 0.2 as *inconclusive*, and *p* > 0.2 as *unrecognized*, the inconclusive rate is 44.1%, the hit rate is 79.6%, and the correct rejection rate is 91.7%.

### Summary of the Threshold

These results will provide reference information to judge the examinee's recognition based on effect size *d* and randomization test *p*. In the known-solution CIT, an examinee would recognize the relevant item if its integrated *d* is more than 0.4 or *p* is < 0.025. In the searching CIT, an examinee would recognize a certain item if its integrated *d* is more than 0.6 or *p* is < 0.01. The *d* value evaluates the response difference quantitatively, whereas the *p*-value evaluates the difference stochastically. Therefore, we would do well to consider both the *d* and *p*-values when judging the examinee's recognition.

Of course, before applying these thresholds to CIT in the field, we must verify them with other datasets. We believe that the proposed statistical judgment methods can be applied to the field datasets, because autonomic responses are essentially the same between laboratory and field CITs (13, 36). However, the magnitude of response differences is sometimes larger in the field than in the laboratory (13). We must therefore confirm whether the thresholds proposed above have sufficient discrimination performance when we apply them to the field datasets.

## Reducing Inconclusive Cases

Although the above section shows high discrimination performance using *d* and *p*-values, it also demonstrates that the inconclusive rates were relatively high, particularly in the searching CIT. To reduce the number of inconclusive cases, we would have to add new measures to the current autonomic measures (2). Recent studies have indicated that facial information, such as eye movement, pupil size, blinks, and facial skin temperature, are promising as new CIT measures (37–41). Some facial information can be recorded using current polygraph devices in Japan (42), but can also be remotely sensed by camera. Remote sensing can dramatically reduce the discomfort of attaching sensors to the examinee. In contrast, voice information obtained by the examinee's responses to each item has rarely been analyzed (43), and could be recorded without attaching sensors. Adding these remote sensing techniques is a new direction in the use of CIT in the field. However, it is important to pay attention to how the examiner informs the examinee about physiological recordings that he or she cannot perceive.

## Conclusion

Although many people think of the polygraph as a deception detection technique, the polygraph based on the CIT should be regarded as memory detection technique. The CIT can reveal what an examinee knows and what he or she does not know about a crime. The CIT can also reveal, through the examinee's memory, new crime-relevant information that the examiner and investigators did not previously know about. Furthermore, the CIT can be used for assessing the credibility of examinees' statements. Correct understanding of the CIT will change the role of the polygraph in criminal investigations. The development of statistical judgment methods will make the CIT more objective and promote its use outside Japan.

The CIT is a scientifically valid method and can reveal how the examinee is related to the crime through his or her memory. Although the CIT has much potential, Japan is the only country in which it has been widely used. We hope that this paper will encourage more practitioners to try CIT in their fields.

## Author Contributions

All authors listed have made a substantial, direct and intellectual contribution to the work, and approved it for publication.

### Conflict of Interest Statement

The authors declare that the research was conducted in the absence of any commercial or financial relationships that could be construed as a potential conflict of interest.

## Footnotes

^1^We chose these horizontal axis ranges of Figure 3 considering the following two points: (1) The range should not be too wide to observe the change of the ratio of the recognizing/unrecognizing examinees according to the increase of the *d*/*p* values; (2) The range also should not be too narrow to include a sufficient number of examinees.
